# Mutagenesis and Resistance Development of Bacteria Challenged by Silver Nanoparticles

**DOI:** 10.1128/aac.00628-22

**Published:** 2022-09-12

**Authors:** Kun Wu, Haichao Li, Xiao Cui, Ruobing Feng, Weizhe Chen, Yuchen Jiang, Chao Tang, Yaohai Wang, Yan Wang, Xiaopeng Shen, Yufei Liu, Michael Lynch, Hongan Long

**Affiliations:** a Institute of Evolution and Marine Biodiversity, KLMME, Ocean University of China, Qingdao, China; b Laboratory for Marine Biology and Biotechnology, Qingdao Pilot National Laboratory for Marine Science and Technology, Qingdao, China; c College of Life Sciences, Anhui Normal University, Wuhu, Anhui, China; d Key Laboratory of Optoelectronic Technology & Systems, Chongqing University, Ministry of Education, Chongqing, China; e Biodesign Center for Mechanisms of Evolution, Arizona State University, Tempe, Arizona, USA

**Keywords:** metallic nanoparticles, antimicrobial agents, drug resistance, experimental evolution, environmental mutagenesis

## Abstract

Because of their extremely broad spectrum and strong biocidal power, nanoparticles of metals, especially silver (AgNPs), have been widely applied as effective antimicrobial agents against bacteria, fungi, and so on. However, the mutagenic effects of AgNPs and resistance mechanisms of target cells remain controversial. In this study, we discover that AgNPs do not speed up resistance mutation generation by accelerating genome-wide mutation rate of the target bacterium Escherichia coli. AgNPs-treated bacteria also show decreased expression in quorum sensing (QS), one of the major mechanisms leading to population-level drug resistance in microbes. Nonetheless, these nanomaterials are not immune to resistance development by bacteria. Gene expression analysis, experimental evolution in response to sublethal or bactericidal AgNPs treatments, and gene editing reveal that bacteria acquire resistance mainly through two-component regulatory systems, especially those involved in metal detoxification, osmoregulation, and energy metabolism. Although these findings imply low mutagenic risks of nanomaterial-based antimicrobial agents, they also highlight the capacity for bacteria to evolve resistance.

## INTRODUCTION

Antibiotics, one of the greatest discoveries of modern biology, have been protecting countless humans from the infections of pathogenic microorganisms. In the early 1930s, bacterial infections killed nearly 300,000 annually, ~22% of total deaths in the United States (1). After the clinical application of antibiotics in the late 1930s, the mortality from severe infections, such as bacterial meningitis and endocarditis, declined by 60 to 75%, and life expectancy has thus increased significantly (2). However, antibiotics are also a double-edged sword for humans. With the misuse or abuse of antibiotics, morbidity and mortality resulting from infections with resistant microorganisms have increased dramatically, making antibiotic resistance, especially multidrug resistance, one of the most health-threatening events globally (3, 4). The declining efficacy of current treatments and the resultant increase in multidrug-resistant (MDR) pathogens constitute a serious worldwide public health problem (3, 5).

As an effective antimicrobial agent, silver has been widely used since ancient times, but its usage declined with the popularity of antibiotics (6–11). With the emergence of MDR microorganisms and the development of nanotechnology, silver nanoparticles (AgNPs) have drawn considerable attention due to their excellent antibiotic effects: AgNPs are exceptionally broad-spectrum antimicrobial agents which can eliminate pathogens ranging from viruses to bacteria to fungi (10, 12–16). Moreover, AgNPs can eradicate biofilms, which are the culprit of chronic infection and MDR strains (17, 18). AgNPs also have diverse action mechanisms, including destruction of the cell wall or membrane, destabilization of ribosomes or other functional proteins, interaction with DNA, and formation of free radicals (17, 19–23). Furthermore, AgNPs can be more stable and have long-lasting antimicrobial effects compared to traditional antibiotics, especially those with the corona layers, i.e., biological coating or stabilized with polyethylene glycol (PEG), polyvinyl pyrrolidone (PVP), and citrate, although their stability can also be highly dependent on their biological environments as well as their particle shape (e.g., spherical AgNPs are typically more stable than their anisotropic counterparts) (24–27). In particular, one exposure experiment of PVP-stabilized AgNPs demonstrated that there was no significant change in shape, aggregation, or dissolution and only a slight decrease in concentration even after 21 days (28). In contrast, most traditional antibiotics, such as ceftazidime and ceftriaxone, lose >10% of the initial concentration at room temperature after 2 to 3 days (29).

Because of their excellent antimicrobial properties, AgNPs are used for a variety of medical applications, such as wound dressings, dental materials, water filters, and so on (30–33). Global production of AgNPs is ~500 tons annually and will reach ~800 tons by 2025 (34, 35), occupying ~25% of total nanomaterial production (36, 37). With the development and application of silver nanotechnology, sublethal levels of AgNPs are widely found in aquatic environments; for example, the predicted concentration in water for 2020 is 225 to 1,799 ng/L (36). It is known that resistance development is a complicated genetic and evolutionary process involving the equilibrium between selection and mutations, and some mutagenic antimicrobial agents can also induce resistance mutations (38). The high stability and long-lasting antimicrobial effects of AgNPs exert more risks to the environment, and the environmental risks of AgNPs, such as microbial mutagenicity, remain to be evaluated. Moreover, bacteria are known to gain resistance to AgNPs through the regulation of osmotic pressure or transcriptional factors, efflux pumps, etc. (39–44), but how resistance generates and evolves remains unclear, and clinical strategies against AgNPs resistance are still lacking (41, 42).

**FIG 1:**
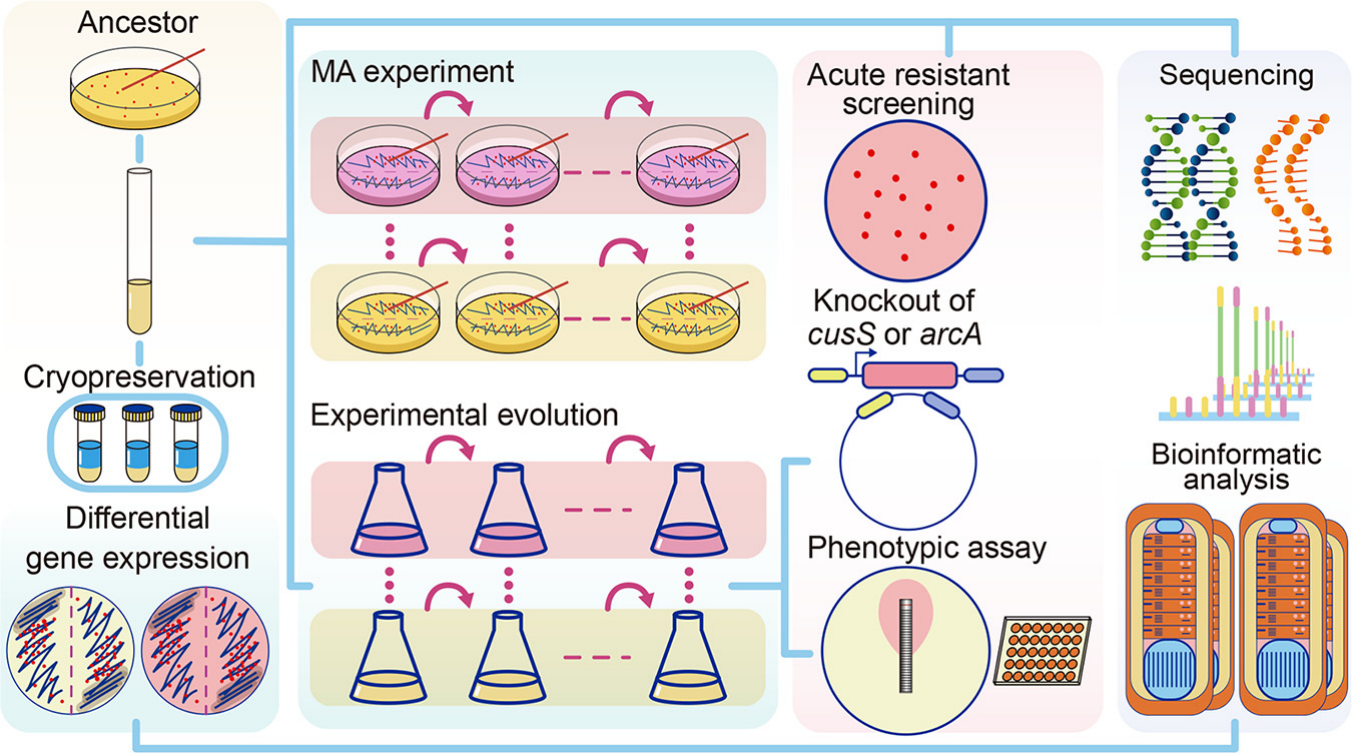
Experimental design.

Here, using the model bacterium Escherichia coli MG1655, we applied mutation accumulation (MA) experiments combined with deep whole-genome sequencing to study the mutagenic effects of AgNPs. During MA, hundreds of replicate lines from a single cell were repeatedly streaked for thousands of generations such that the strong population bottlenecks for each cell line minimized selection, allowing even highly deleterious mutations to accumulate and providing unbiased estimates for genome-wide mutation rates and their molecular spectra at single-nucleotide resolution. Differential gene expression analyses upon AgNPs treatments with sublethal or bactericidal doses were also performed to help reveal the action mechanisms of AgNPs. Finally, we did experimental evolution experiments and gene editing to study the emergence of resistance development mechanisms upon AgNPs treatment (Fig. 1).

## RESULTS

**FIG 2:**
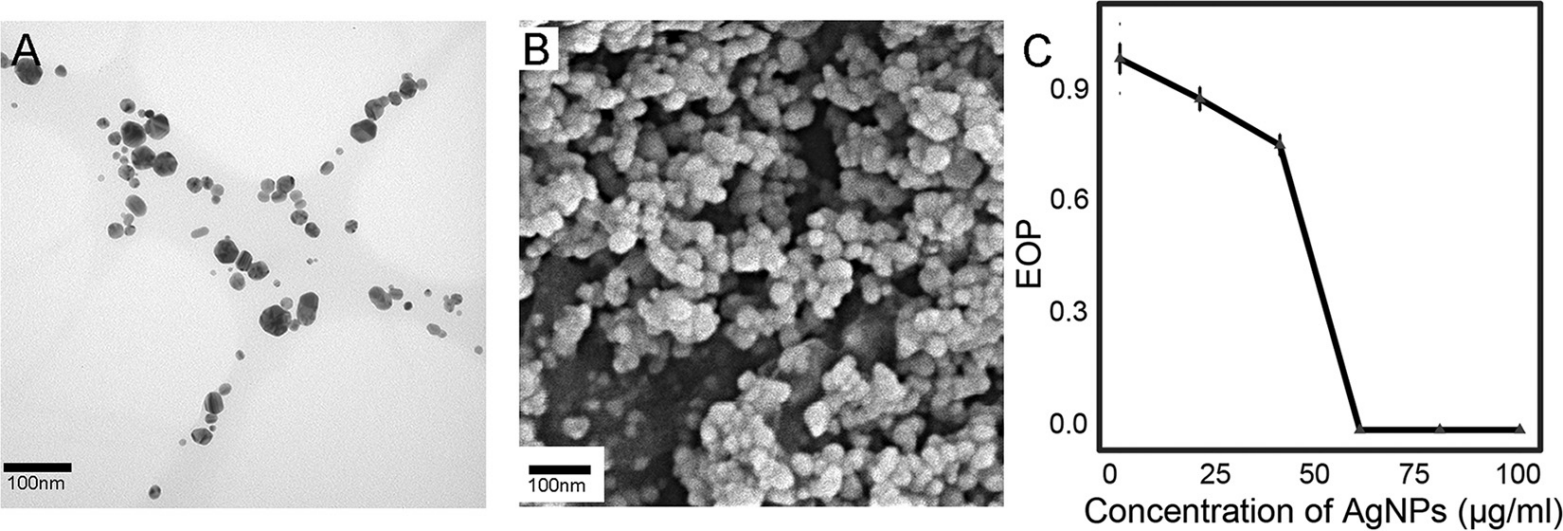
Characteristics of AgNPs in this study. (A, B) Micrographs of TEM and SEM, respectively. (C) Survival curve of E. coli under AgNPs treatment. Error bars represent standard deviation.

We synthesized AgNPs by biological reduction and measured the particles’ size, shape, pH, zeta potential, and thermostability (Fig. 2; see Fig. S1A through H in the supplemental material). The minimum inhibitory concentration (MIC)—the lowest concentration preventing visible growth of bacteria—of the model strain Escherichia coli MG1655 to AgNPs were also quantified (Fig. 2C). Based on the MIC, 60 μg/mL of AgNPs were used for MA, transcriptome sequencing (RNA-seq), and experimental evolution experiments unless otherwise specified. We chose 60 μg/mL of AgNPs for a sublethal concentration as high as possible so that maximum selection pressure or significant gene expression could be achieved upon AgNPs treatment.

### Characterizing AgNPs made in the lab.

Lab-made AgNPs were characterized by UV-visible (UV-vis) spectrometry, transmission electron microscopy (TEM), and scanning electron microscopy (SEM). The absorption peak of AgNPs detected by the UV-vis spectrum is at ~415 nm, which is highly consistent with previous reports (45, 46) (Fig. S1C). Because the antimicrobial potency of AgNPs is determined by size, shape, concentration, and so on (12, 47), we also measured the sizes of quasispherical AgNPs particles using the TEM micrographs in ImageJ (Fig. 2A), yielding a mean diameter of 18.21 nm (standard deviation [SD], 9.28) (Fig. S1A and B). To further evaluate the physical properties of the AgNPs nanosuspension in the culturing system, we measured the zeta potential, a critical parameter for quantifying the surface charge potential. The mean zeta potential of AgNPs is −15.27 mV (SD, 0.19), demonstrating the negative charge on the AgNPs’ surfaces as expected (Fig. S1D). We further performed the thermostability tests of the AgNPs at melted-agar temperature (60°C) by comparing the size distribution and the zeta potential of AgNPs at 25°C versus 60°C, and there was no significant difference observed; there was no significant difference in UV spectrum and zeta potential after ~24 h as well (Fig. S1B through E and G through J). The unchanged UV absorbance of the LB broth with AgNPs indicated that there was no AgNPs aggregation at least in one culturing cycle of the experiments (Fig. S1K). In addition, because pH is one of the key factors affecting mutational profiles (48), we thus measured the pH of the LB plates with and without AgNPs, finding no significant difference (Table S1; Fig. S1L, chi-square test, *P* ≈ 1). All of the above-described results demonstrate the regular physical properties and stability of the AgNPs in our study.

In order to determine the MIC of AgNPs in E. coli MG1655, we calculated the efficiency of plating (EOP) for the ancestor strain to estimate the survival rates under AgNPs treatments (Fig. 2C). The EOP of E. coli decreases with AgNPs concentration and drops to zero at 60 μg/mL, *viz.*, the MIC.

### AgNPs treatment dramatically changes gene expression profiles of E. coli.

**FIG 3:**
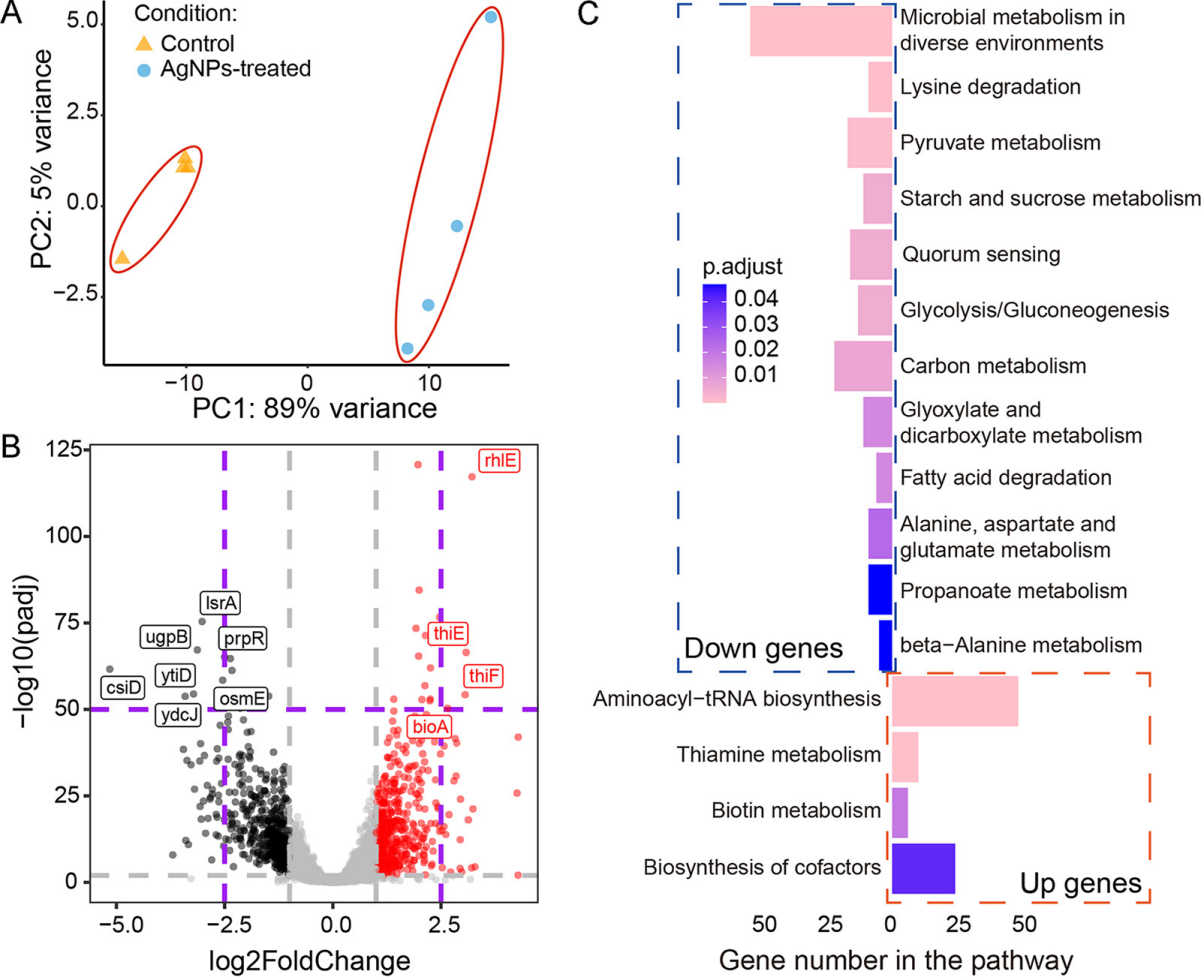
Differential gene expression of E. coli upon AgNPs treatment. (A) PCA plot of clustering based on gene expression. (B) Differential gene expression of AgNPs versus control treatment. Red, black, and gray dots represent significantly upregulated genes, significantly downregulated genes, and not significantly differentially expressed genes, respectively. (C) Upregulated and downregulated genes from KEGG analysis. Up genes and down genes represent the upregulated and downregulated genes under AgNPs treatment versus control.

For systematically revealing the action mechanisms of AgNPs on E. coli, we applied RNA-seq on the control and the treatment and then analyzed differential gene expression (DGE) upon AgNPs treatment by using gene ontology (GO) and KEGG pathway enrichment analyses. After filtering out low-quality reads, a total of 307.85 and 314.35 million clean reads were used for the control and AgNPs treatment for downstream analysis, respectively. We then performed sample-level quality control using principal-component analysis (PCA) and heatmaps and then identified 450 upregulated and 462 downregulated genes in the AgNPs treatment compared with the control, leading to 4 and 12 KEGG pathways, respectively (Fig. 3; Fig. S2).

Based on KEGG analysis, AgNPs treatment substantially slowed the overall metabolism of the cells, especially the microbial metabolism in diverse environments (54 downregulated genes, including substance modification and catabolic process, energy production and transmission, and so on) and quorum-sensing (16 downregulated genes) pathways (Fig. 3C). Among them, *prpR*, *csiD*, *lsrA*, *ugpB*, *osmE*, *ydcJ*, *ytiD*, *prpR*, and *csiD* are the most downregulated ones (Fig. 3B). These are mostly canonical genes involved in the transcriptional regulation of propionate catabolism, catalyzing hydroxylation of glutarate (GA), and l-2-hydroxyglutarate (L2HG). Interestingly, three genes, *ydcJ*, *osmE*, and *ytiD*, previously not annotated to any specific functions, might be associated with certain metabolic activities upon antimicrobial agent treatment. Further explorations of their gene functions are needed. All of the above observations suggest that AgNPs may inhibit intercellular information exchange and signal sensing of target cells and potentially reduce the formation of biofilms.

In contrast, biosynthesis of the aminoacyl-tRNA synthetases and cofactors and the metabolism of thiamine and biotin were upregulated in expression, with 164 genes involved (Fig. 3B and C), and four genes, *bioA*, *thiF*, *thiE*, and *rhlE*, having the most extreme expression elevation. The first three genes are transferases involved in the catalytic biosynthesis of thiamine and biotin, such as 7,8-diaminopelargonic acid (DAPA) and thiamine monophosphate (TMP). Thiamine and biotin are two types of vitamin B, essential for converting certain nutrients into energy. *rhlE* is an RNA helicase involved in ribosome assembly and improving ribosome biogenesis. Further exploration is needed to determine whether the upregulation of these biological functions reflects partial compensation for the decreased overall metabolism.

The SOS response pathway is a global response to DNA damage in which DNA repair and low-fidelity DNA polymerases are induced, leading to elevation of the genome-wide mutation rate (38). Comparing the gene expression under the AgNPs treatment with the control, we did not find significant elevation in most genes of the SOS response pathway and low-fidelity DNA polymerases (Table 1; Table S2). This shows that sublethal AgNPs treatment does not induce the SOS response, further implying that AgNPs might not damage DNA. Thus, we speculate that AgNPs do not affect bacterial genome stability and may not elevate genome-wide mutation rates. To further test this, we performed the following MA experiments, combined with deep whole-genome sequencing and mutation analyses, to assess the mutagenic effects of AgNPs at single-nucleotide resolution and whole-genome scale (38).

**TABLE 1:** Information about genes with high mutation hits or in the SOS response pathway^*a*^

Gene name	Expression change in log_2_ fold change (AgNPs vs control)	*P* value	Change	Expt(s)	Function
*narU*	−1.21	2.46 × 10^−9^	Down	MAC	Nitrate assimilation
*alaW*	0.99	5.10 × 10^−3^	NoDiff	MAC	Aminoacyl-tRNA synthetase
*argG*	−0.13	1.62 × 10^−1^	NoDiff	MAC	Amino acid biosynthesis
*pheU*	2.12	1.09 × 10^−7^	Up	MAC	Uncharacterized protein
*cueO*	1.4	2.55 × 10^−7^	Up	MAA	Copper efflux oxidase
*ynbG*	0.67	1.42 × 10^−1^	NoDiff	MAA	Uncharacterized protein
*isrC*	0.24	3.41 × 10^−1^	NoDiff	MAA	Acyltransferase
*ompR*	−1.04	1.18 × 10^−25^	Down	ARS	Osmoregulation
*arcA*	−1.19	3.81 × 10^−32^	Down	EE	Aerobic respiration control protein
*cusS*	0.70	1.15 × 10^−8^	NoDiff	MAA, EE, ARS	Cu2^+^ or Ag^+^ response regulation
*cusR*	0.61	6.35 × 10^−6^	NoDiff	MAA, EE, ARS	Cu2^+^ or Ag^+^ response regulation
*polB*	−0.37	3.37 × 10^−3^	NoDiff		Low-fidelity DNA polymerases
*dinB*	−0.21	1.07 × 10^−1^	NoDiff		Low-fidelity DNA polymerases
*umuD*	−0.64	3.02 × 10^−3^	NoDiff		Low-fidelity DNA polymerases
*umuC*	−0.12	2.39 × 10^−1^	NoDiff		Low-fidelity DNA polymerases
*uvrA*	0.36	3.08 × 10^−3^	NoDiff		SOS response pathway
*recA*	0.24	2.95 × 10^−2^	NoDiff		SOS response pathway
*uvrB*	−0.68	3.17 × 10^−5^	NoDiff		SOS response pathway
*uvrD*	0.06	5.63 × 10^−1^	NoDiff		SOS response pathway

a MAC, MAA, ARS, and EE represent MA-control, MA-AgNPs, acute resistance screening, and experimental evolution, respectively. NoDiff, no difference. The cutoff *P* value is 0.01.

### Sublethal AgNPs concentrations do not alter the genomic rate or molecular spectrum of mutations in E. coli.

In total, from a single-cell ancestor, two groups of MA lines were initiated, each with 200 replicate lines, with or without AgNPs treatment. Each of the control MA lines experienced ~1,099 cell divisions on average and ~1,112 for the AgNPs treatment, respectively. Illumina PE150 genome sequencing was done for all final lines. After filtering out cross-contaminated and low-coverage lines, 142 and 172 MA lines for the control and the treatment were used in the final analyses, respectively (Tables S3 to S10). All MA lines were sequenced at a high depth of coverage and mapping rate (Tables S3 and S7).

Despite the design of the MA experiments, sublethal AgNPs treatment might bias the mutation rate and spectrum by selecting for strong resistance mutations. We thus calculated the ratio of nonsynonymous-to-synonymous substitutions but found no significant difference from the neutral expectation in either the control (Pearson’s chi-square test, *χ^2^* = 0.48, degrees of freedom [df] = 1, *P = *0.49) or the AgNPs treatment (*χ^2^* = 5.06 × 10^−20^, df = 1, *P = *1.00) (Table 2; Tables S4 and S8). In addition, the *N_e_* (effective population size, the critical parameter in population genetics, defined as the equivalent number of individuals in one idealized population) of both the control and AgNPs treatment was around 14, indicating that drift dominated selection even in the AgNPs-treated MA.

**TABLE 2:** Counts and proportions of different BPSs from the control and treatment^*a*^

Category	Count/proportion of:	
	Control	Treatment
Intergenic regions	24/0.23	19/0.19
Coding region	81/0.77	79/0.81
Overlap	1/0.01	0/0
Synonymous	17/0.21	20/0.25
Nonsynonymous	63/0.78	59/0.75
Transitions	54/0.51	57/0.58
A:T→G:C	19/0.35	23/0.40
G:C→A:T	35/0.65	34/0.60
Transversions	51/0.49	41/0.42
A:T→T:A	13/0.25	14/0.34
A:T→C:G	18/0.35	13/0.32
G:C→C:G	5/0.10	2/0.05
G:C→T:A	15/0.29	12/0.29
Insertions	7/0.50	7/0.44
Deletions	7/0.50	9/0.56

a Count is the total number of BPSs in the MA lines; proportion refers to the proportion of the BPSs in the category out of the total BPSs in the MA lines.

**FIG 4:**
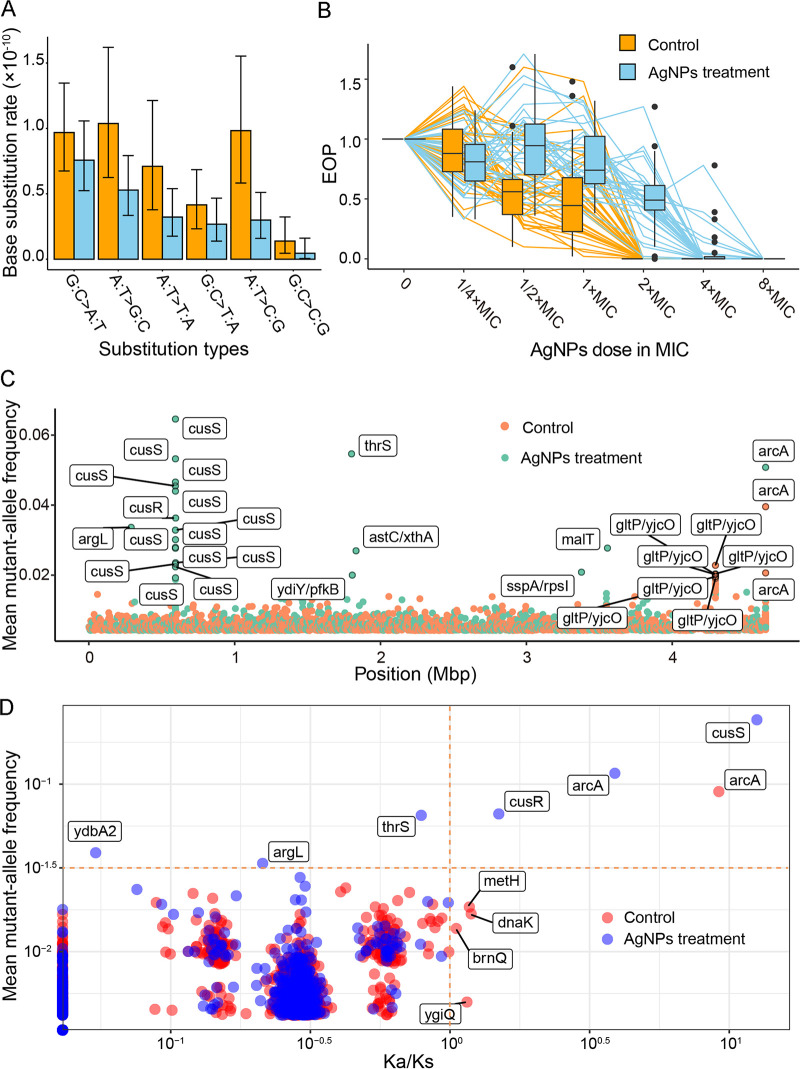
Mutation (A), resistance (B), and evolutionary patterns (C, D). (A) Mutation spectrum of MA lines from the control (orange) and AgNPs treatment (blue). (B) Survival curves of single colonies randomly chosen from experimental evolution lines. (C) Mean mutant allele frequencies of genomic sites in the control or the AgNPs-treated experimental evolution lines. The genes with the top 30 mutation frequencies are marked. Note that the symbols for identical genes (for example, *cusS*, *arcA*, and *gltP*/*yjcO*) actually represent different sites, which seemingly occur at the same site due to a genome-wide scale. (D) Mean mutant allele frequencies of genes in each group (control or AgNPs-treated) and *K_a_*/*K_s_* of each gene.

In total, 105 and 98 base pair substitutions (BPSs) were accumulated in the control and the AgNPs treatment, leading to mutation rates of 1.45 × 10^−10^ (95% Poisson confidence interval [CI], 1.19 × 10^−10^ to 1.76 × 10^−10^) and 1.11 × 10^−10^ (95% CI, 8.68 × 10^−11^ to 1.31 × 10^−10^) per nucleotide site per cell division, respectively. The mutation spectra do not differ significantly, with similar transition-to-transversion ratios (1.06 versus 1.36 in the control and AgNPs treatment, *χ^2^* = 0.68, df = 1, *P = *0.41) (Table 2; Fig. 4A). Furthermore, we also parsed out mutations at 4-fold degenerate sites, where any mutations do not change the encoded amino acids and are thus not under selection, and the mutation rates were still not significantly different between the control and the AgNPs treatment (1.32 × 10^−10^; 95% CI, 7.71 × 10^−11^ to 2.12 × 10^−10^, versus 1.24 × 10^−10^; 95% CI, 6.60 × 10^−11^ to 2.12 × 10^−10^). We also detected 14 and 16 small indels in the control and the treatment, respectively, yielding indel rates of 1.94 × 10^−11^ (95% CI, 1.06 × 10^−11^ to 3.25 × 10^−11^) (Table S5) and 1.81 × 10^−11^ (95% CI, 1.03 × 10^−11^ to 2.94 × 10^−11^) (Table S9), as well as insertion/deletion ratios of 1.00 and 0.79 (*χ^2^* = 0, df = 1, *P = *1). Thus, sublethal AgNPs concentrations do not change genome-wide mutational features of target cells.

### Rapid acquisition of resistance upon treatment with high AgNPs concentration.

Although AgNPs do not change bacterial mutational patterns, resistance to AgNPs could arise from selection of cells carrying resistance mutations and/or other advantageous traits for clonal expansion. We then screened 75 resistant colonies, each of which was selected by treating ~1 × 10^5^ ancestral E. coli cells at an optical density (OD) of ~1 on LB agar plates with 480 μg/mL of AgNPs (8× MIC). In total, 15 single-nucleotide polymorphism (SNP) sites were detected in three genes, *cusS*, *cusR*, and *ompR* (Table 1, Fig. S3A, and Table S11), belonging to two-component regulatory systems.

*cusS* and *cusR* are known to be associated with silver resistance: the Cus system is the first identified silver resistance system that can transport silver ions into the extracellular space (40, 49). *ompR* is one member of the two-component system EnvZ/OmpR involved in osmoregulation (50), which may decrease the infiltration of AgNPs into cells. These results demonstrate that upon acute treatment with high AgNPs concentration, populational resistance to AgNPs is mainly associated with mechanisms of silver ion resistance and/or osmoregulation.

We also iterated genes with significantly elevated mutations in the mutation accumulation lines treated with 60 μg/mL AgNPs. After the Poisson test with Bonferroni correction, five genes (*cusS*, *cusR*, *cueO*, *ynbG*, and *isrC*) were found to have significantly elevated mutation rates, probably selected for conferring resistance to AgNPs (Table 1; Tables S2 and S10). This confirms the above-described resistance mechanisms even at the sublethal AgNPs level.

### Resistance gain of populations upon sublethal AgNPs treatment in liquid medium.

To determine whether bacteria can gain resistance at sublethal treatment over a longer time span through the above-described two-component-system resistance mutations or alternative mechanisms, with strong selection present, we initiated two groups of parallel lines cultured in liquid LB (24 in total) from a single-cell ancestor. One group of 12 lines was cultured in 100 mL LB medium with 0.1 mL transferred daily (evoLL1 to evoLL12), and the other group of 12 lines in 100 mL LB medium with 60-μg/mL AgNPs was transferred similarly (evoLS1 to evoLS12). The mean cell densities of the control and the treatment over the entire experiment were 2.75 × 10^9^/mL and 2.82 × 10^9^/mL (Fig. S3B), respectively (*t* test, *P = *0.35) (density was measured by CFU every 15 transfers). The cells treated with the sublethal AgNPs concentration might take a much longer time for resistance mutations to become dominant or fixed than the above-described lethal treatment. We thus transferred each line 40 times and then plated the final evolved lines onto LB agar, randomly chose and genome sequenced three colonies from each line, and measured their MICs (Tables S12 and S13; Fig. 4B). Resistance of the final evolved lines in both the control and AgNPs treatment increased dramatically (MIC of the final control lines, 2× ancestral MIC, SD = 0; 5.5× ancestral MIC, SD = 1.94× for the AgNPs-treated final lines). Even the final evolved control lines gained resistance of 2× ancestral MIC. The resistance increase in the final evolved control was not caused by contamination of AgNPs or other lines because various precaution procedures and sufficient training of operators have been applied to avoid cross-contamination or mistakenly adding AgNPs to the control lines (see Materials and Methods for details); in addition, all 12 final AgNPs-treated experimental evolution lines accumulated *cusS* or *cusR* SNPs, while, in contrast, in the control experimental evolution lines, we did not find a single SNP in the *cusS* or *cusR* genes of any line by genome sequencing either the randomly picked colonies or the populations (Table S13; Fig. S4 and S5). For the final evolved AgNPs-treated lines, resistance ranged from about 4 to 8× the ancestral MIC. After sequencing and mutation calling of single colonies from each evolved experimental evolution line, we found that 41.67% of SNPs in the coding regions of the control lines are in *arcA* or *arcB* (6.06% in AgNPs-treated lines); 24.24% of coding-region SNPs are in *cusS* for the treatment (0% in control; Table S13). ArcA/ArcB is also a two-component regulatory system involved in energy generation (51).

**FIG 5:**
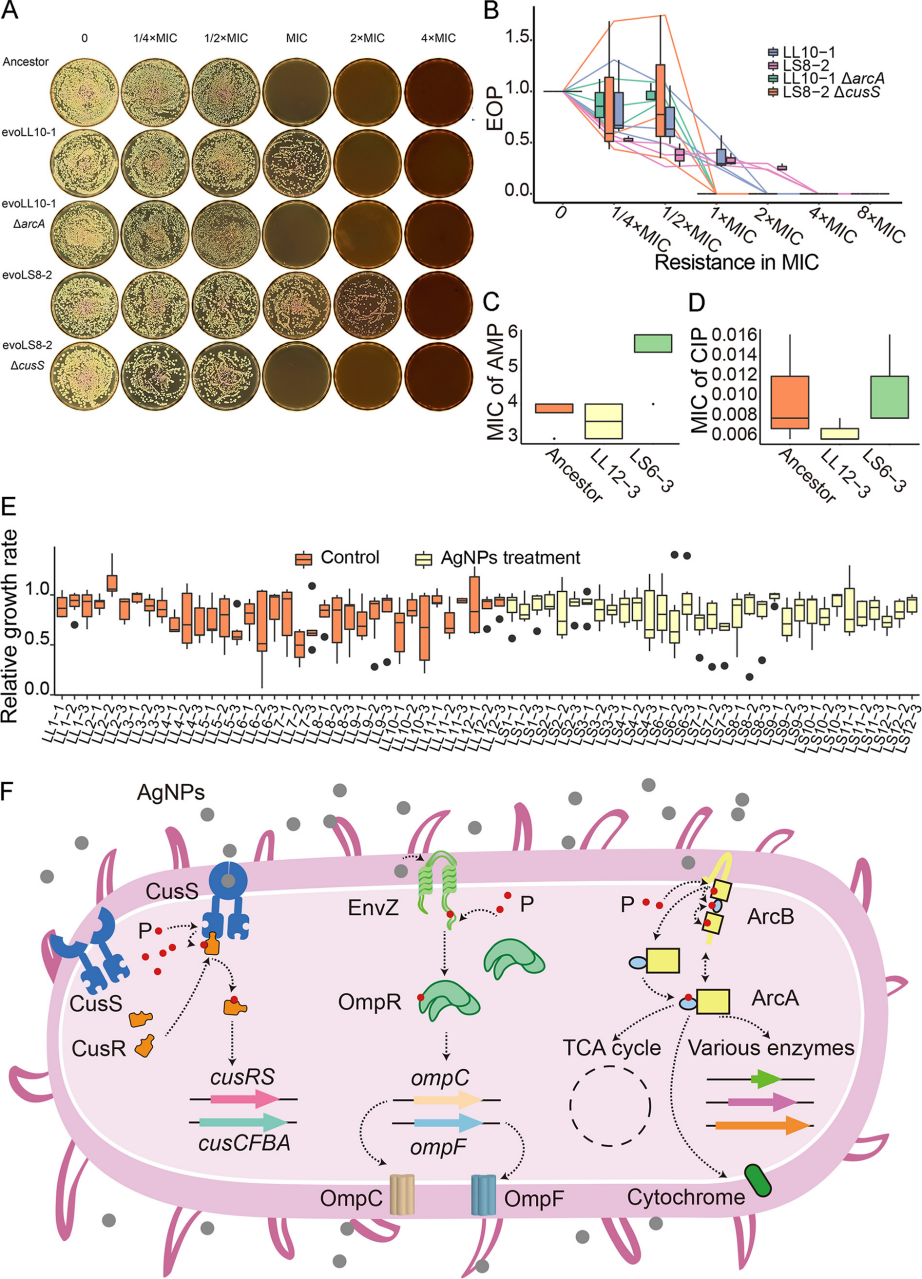
Resistance tests and growth rates. (A, B) Tests of AgNPs resistance, given as MICs, for the ancestor, evoLL10-1, evoLL10-1 Δ*arcA*, evoLS8-2, and evoLS8-2 Δ*cusS*. (C, D) Resistance tests of strains with different genetic backgrounds, ampicillin (AMP), and ciprofloxacin (CIP). (E) Growth rates of single clones randomly chosen from the final experimental evolution lines. Growth rate measurement followed Wang et al. (112). The growth rates were assayed in the absence of AgNPs, normalized by dividing the growth rate of the ancestor. (F) Resistance mechanisms of bacteria upon AgNPs treatment, based on this and previous studies (49–51). P, phosphate group.

To understand the evolution of the key genes associated with the aforementioned AgNPs resistance, we did population sequencing at ~98× and 105× depth of coverage for the control and the AgNPs treatment and analyzed mutations by using breseq (v0.35.7) at an allele frequency of >5% (Tables S14 and S15). We detected 2,000 and 1,174 variants distributed in 1,532 and 935 genes for the control and the AgNPs treatment, respectively (Tables S15 and S16). We also compared the ratio of the number of nonsynonymous substitutions per nonsynonymous site to the number of synonymous substitutions per synonymous site (*K_a_*/*K_s_*), which is used to evaluate selection assuming the latter is neutral (Fig. 4D; we calibrated the *K_a_*/*K_s_* of genes with 0 synonymous mutations with a simple regression method, shown in Fig. S3C). Positive selection appears to increase the frequency of mutations of resistance genes such as *cusS*, *cusR*, and *arcA* (Pearson’s product-moment correlation, *r *= 0.79, *P = *0.21) and leads to convergent evolution of the genes in parallel lines (Fig. S4 and S5). These results confirm again the key role of the Cus system in the resistance development of AgNPs-treated bacteria. To further evaluate this hypothesis, we knocked out the gene *cusS* in evoLS8-2, which has a single mutation (Table S13), and found that the resistance decreased to the same level as the ancestor (Fig. 5A and B), confirming the association between the mutation and AgNPs resistance.

As shown by the above-described colony and population sequencing of both the control and the AgNPs-treated experimental evolution lines, the positively selected gene *arcA* has high mutation hits (Fig. 4C and D). This indicates that *arcA* might be pleiotropic in affecting both metabolism and AgNPs resistance. The *arcA* mutants were possibly selected for energy generation during clonal expansion and AgNPs resistance even in the untreated lines. Taking account of the MIC increase (2× ancestral MIC; multiple precautions were applied during transfers to avoid the control lines being contaminated by AgNPs; see details in Materials and Methods) and the high frequency of *arcA* mutations (48%; Table S13) in the control lines, we hypothesize that ArcA mutants elevate energy generation during growth in the untreated liquid medium and also increase MIC to compensate the reduced metabolism by AgNPs. To verify this, we knocked out the mutant *arcA* of evoLL10-1, a subline established from a single colony of the control experimental evolution line evoLL10 (2× ancestral MIC), and found that the resistance did decrease to the ancestral level (Fig. 5A and B). Moreover, mutant lines carrying the *arcA*/*arcB* SNPs (from single colonies isolated from the final evolved control experimental evolution lines) did show elevated median resistance (0.42 versus 0.26 for lines with or without *arcA*/*arcB* mutations, respectively, estimated by the EOP at 2× ancestral MIC; Fig. S6A), though not significantly (Wilcoxon rank-sum test, *P = *0.11). All of the results indicate that *arcA* mutations increase the resistance to AgNPs. We further compared the growth rates of the ancestor, evoLL10-1, and evoLL10-1 Δ*arcA* in LB broth without AgNPs (0.58 per hour, SD = 0.02; 0.53 per hour, SD = 0.02; 0.40 per hour, SD = 0.01, respectively [Fig. S6B and C]; evoLL10-1 carries only one *arcA* mutation, genomic position 4,639,996 G→T, compared with the ancestor and was established from a single colony of the evolved control line evoLL10 [Table S13]). We found that the growth rate of evoLL10-1 decreases to 0.92× that of the ancestor, a direct demonstration of the fitness cost of the *arcA* mutation (Fig. S6B). The growth rate of evoLL10-1 Δ*arcA* decreases even further to ~0.70× that of the ancestor. This supports the pleiotropic effects of *arcA*, as removing the mutant *arcA* not only eliminated the resistance mutation but also other functions associated with *arcA*, *viz.*, the fitness costs associated with the loss of other functions overwhelm the recovered fitness by the loss of the resistance mutation.

We also observed that when the cell density is quite high (~10^8^/mL), the ancestral cells treated even with extremely high AgNPs concentration, i.e., 960 μg/mL or 12× ancestral MIC, did not form single colonies but a lawn. We speculate that high population density might be associated with enhanced resistance, a sign of quorum sensing. To confirm this, we chose genes with mutation frequency higher than 0.09 upon AgNPs treatment, and we performed GO analysis. Surprisingly, only one pathway, quorum sensing (QS), was enriched, which included six genes (*oppF*, *pdeR*, *ydcZ*, *sdiA*, *rcsA*, and *ftsY*) functioning in DNA binding and transcription involving dozens of mutational sites (Table S15). Thus, QS of bacteria was an important response mechanism during AgNPs treatment. Even though RNA-seq shows that AgNPs reduce QS expression (Fig. 3C), QS does play an important role in population-level resistance. Future work will address the complicated issues with respect to the associations between mutations and QS.

As shown above, AgNPs are antimicrobial agents with diverse action mechanisms. An interesting question is whether resistant bacteria developed from AgNPs treatment would also gain resistance to other antibiotics. We thus chose the subline evoLS6-3 (established from a single colony of the final evolved treated line evoLS6) with the largest resistance increase (8× ancestral MIC), the final evolved control line evoLL12-3, and the ancestral line to measure resistance to ampicillin (AMP) and ciprofloxacin (CIP), which act by inhibiting cell wall synthesis and DNA replication, respectively. We did not find any significant resistance in the AgNPs-treated line (Fig. 5C and D). Interestingly, for evoLS6-3, there are only two base substitution mutations in noncoding regions detected (Table S13). Whether these mutations increase resistance by falling into regulating motifs needs further study in the future. In addition, we also randomly chose eight single colonies from each evolved control or treated line, assayed the growth rate in the absence of AgNPs, and found no significant difference (Fig. 5E). These demonstrate that AgNPs treatment does not lead to multidrug resistance in the beta-lactam and fluoroquinolone antibiotics, possibly also the case for other antimicrobial nanomaterials, further alleviating concerns about their environmental risks.

## DISCUSSION

AgNPs are long-lasting antimicrobial agents, mostly because of their high metal stability compared with traditional antibiotics. While more AgNPs products are being developed and consumed, such as daily necessities and personal items, they could pose a potential threat to human health by possibly inducing or selecting resistant pathogens. In this study, we found no mutagenic effects of the silver nanoparticles, giving direct support for the low environmental risk of the application of this genuinely broad-spectrum antimicrobial agent. The ability to reduce QS is another advantage of AgNPs over traditional antibiotics. AgNPs treatment does not trigger multidrug resistance either, another hallmark of preferred antimicrobial agents. Nonetheless, E. coli can still gain AgNPs resistance, mainly through selection for mutations in multiple two-component regulatory systems (Fig. 5F). To reveal the evolutionary dynamics in higher resolution, future experiments with bacterial populations treated for longer time spans and with intermediate time points are needed; those treated with AgNPs products in clinical scenarios would be even more interesting for studying pathogen evolution or resistance mechanisms. AgNPs are also known to catalyze reactions or oxidize chemicals, such as catalyzing the reaction of borohydride reducing various dyes (52) and oxidation of benzene (53), so whether AgNPs in these scenarios indirectly affect genome stability and then elevate the emergence of drug-resistant bacteria is worth further research.

Whether AgNPs damage DNA of target bacteria, *viz.*, mutagenic, is highly controversial. One study shows that AgNPs can interact with the phosphate groups of DNA and cause irreversible destruction (21), and Ag(I) ions can form a duplex comprising three imidazole-Ag^+^ imidazole base pairs in genomic DNA (54). But others argue that DNA is not affected by AgNPs treatment in bacteria or animals (20, 55, 56). In our experimental systems, we did not detect any gene expression associated with DNA damage, such as the SOS response, upon AgNPs treatment (Tables 1 and 2; Fig. 4A). Most conspicuously, AgNPs do not accelerate the genome-wide mutation rate of bacteria, contrary to the scenario with some antibiotics, such as fluoroquinolones (38, 57, 58), a sign of low environmental risk of AgNPs when released in nature. Moreover, previous studies also showed that AgNPs-treated cells would produce extra reactive oxygen species (ROS) (59, 60), but our study does not support this: in the AgNPs treatment, most genes involved in defense against oxidative stress were not upregulated (see Table S2 in the supplemental material, “Functions” column, “Defense against oxidative stress”); we did not find a significant increase in the mutation rate of oxidative damage-associated mutations either, such as G:C→T:A transversions (Fig. 4A) (61). In addition to AgNPs, more metal nanoparticles, such as gold or copper ones, have also been applied to various aspects of our lives, for example, biomedicine, biosensing, and so on (62–64), and their environmental risks could be tested with the research framework designed in this study.

Some researchers have found that cells can gain resistance to silver nanoparticles or ions through complex mechanisms related to osmotic pressure regulation; transcription regulation factors; the *sil* system, including *silCBA*, *silP*, *silE*, and *silF*; and/or copper-transporting efflux systems involving *ompA*, *ompB*, *ompF*, and *ybdE* (39–41, 43, 44). In our E. coli lines treated with sublethal AgNPs in liquid LB over ~40 days, these reported resistance genes do not show elevated mutation frequency or signs of positive selection (Fig. 4D; Table S15), while the point mutations in *cusS*, *cusR*, and *arcA* are significantly and positively selected by sublethal AgNPs treatments, similar to the results of Graves et al. (42) and Stabryla et al. (65). These results imply that appearance of resistance mutations may be time or dose dependent. To note, there is no association between SNP number and expression level for *cusS*/*cusR* genes. This could be explained by the fact that AgNPs treatment could select cells carrying SNPs in *cusS*/*cusR* genes for resistance and these SNPs could originate from spontaneous mutations caused by replication errors, escape of the DNA repair systems, oxidation, etc., without altering the expression level. *thrS* is the “housekeeping” gene threonyl-tRNA synthetase, which catalyzes the attachment of threonine to tRNA (66, 67). Even though its *K_a_*/*K_s_* is 0.79 in the AgNPs-treated experimental evolution lines, *thrS* has relatively high mean mutant allele frequency (0.07), and the mutant allele frequency of one *thrS* site (genome coordinate of 1,801,362) even reached 0.66 in the evoLS6 population (Fig. 4D; Table S15). The results suggest that *thrS* could be a potential AgNPs resistance gene, and synonymous mutations might still be under certain selection. *thrS* may speed up protein synthesis and thereby compensate for the decreased catabolism rate caused by AgNPs, i.e., keep the protein catabolism yield by increasing the protein substrates, assuming constant stoichiometry of the protein metabolism. In addition, AgNPs treatment decreases the metabolism of target cells, as inferred by our RNA-seq data set (Fig. 3), and it is also known that slowed metabolism is associated with persistence/tolerance (68). This complicates the understanding of the action mechanisms of AgNPs on bacteria, while it is consistent with previous reports of AgNPs inhibiting the formation of biofilms, which is also associated with tolerance (68–71). Interestingly, such metabolism downregulation by AgNPs treatment also exists in various organisms besides bacteria (72–74), providing ample subjects for further research on resistance, persistence, and tolerance to AgNPs.

Quorum sensing is a biological function involving series of communication circuits for regulating gene expression in response to cell density (75), and it controls several phenotypes such as swarming migration, biofilm formation, virulence, and so on (76–78). It could cause severe chronic diseases or antibiotic resistance in important pathogens, such as Serratia liquefaciens and Vibrio cholerae (78, 79). Fortunately, AgNPs, as one agent downregulating QS (80, 81), significantly reduce the expression of 16 quorum-sensing genes based on our RNA-seq data set, which represent 66.67% of all the 24 KEGG QS genes of prokaryotes (KEGG Orthology database) (Fig. 3C). This is highly consistent with previous toxicology studies which show that AgNPs decrease the expression of virulence factors involved in QS, and they inhibit the formation of biofilms (80, 82). Some antibiotics, such as daptomycin and vancomycin prophylaxis, cannot treat infections of methicillin-resistant S. aureus alone but can work when combined with silver (83). Polymyxin B rapidly penetrates the outer cell membrane, and AgNPs can synergistically enhance the permeabilization of cells (84). These have inspired antibiotic treatment plans by combining antibiotics with AgNPs, where QS and other action mechanisms facilitate efficient and effective antimicrobial therapy (84–86). Combining antibiotics with AgNPs is thus a compelling therapeutic approach that can revive ineffective antibiotics, inhibit multiresistant bacteria, and decrease resistance acquisition (87). Unavoidably, bacteria will still evolve resistance to such therapies mainly through enzymatic inactivation, modification of antibiotic target sites, reducing uptake by changes of membrane permeability or presence of porins, and drug extrusion by efflux pumps (e.g., *cusS* in our study) (88). To overcome these multidrug-resistant bacterial infections, it is urgent to identify more mechanisms of resistance and combine all kinds of technological approaches, such as phage therapy and CRISPRs, in addition to nanotechnology (89). Furthermore, QS is known to be associated with multiple two-component systems (90), and the exact pathways by which AgNPs downregulate QS remain unsolved. The association of the three two-component systems (CusS/CusR, ArcA/ArcB, and EnvZ/OmpR, functioning in AgNPs resistance in this study) with QS is still unknown and worth further study. AgNPs are widely used in biomedical applications, such as implanted medical devices, to prevent implant-associated infections mainly caused by biofilm-forming microorganisms (83, 91, 92). Researchers have developed various methods to prevent infections, including chemical or physical antiadhesive resurface, bactericidal materials or innate host immune molecules, and QS quenchers (92). Applying AgNPs as such topical antiseptics should have a bright future because of their long-lasting effect, low mutagenicity, and QS inhibition, as shown in this study.

It remains a question of whether the antimicrobial effects are conferred through the silver nanoparticles and/or Ag ions. This is because the toxicology of AgNPs is highly complex, as inferred by previous studies, possibly resulting from the uncontrolled physical and chemical properties, e.g., size, shape, zeta potential, and oxidation state (93, 94). Ag ions also have a similar action mechanism to AgNPs such as interaction with thiol, induction of hydroxyl radicals, and effects on enzymes associated with the respiratory chain (95). Our study shows Ag ions’ role as one player, as two of the most significantly elevated genes, *cusS* and *cusR*, function in the transport of Cu/Ag ions, consistent with some previous reports (21, 96), while AgNPs are the main factor inhibiting QS, as shown in this and previous studies (97). Our data support that both metallic and ionic Ag are involved in the antimicrobial actions of AgNPs. Some studies have reported that the increased resistance of AgNPs originates from particle aggregations caused by flagellum-based motility (41, 65), which may be considered a shortcoming compared with Ag ions. Nevertheless, AgNPs can still inhibit the nonmotile bacteria and are effective as coated materials applied in medical devices (65). Future metal nanoparticle design needs to reduce or avoid the biologically induced aggregation of AgNPs.

In summary, all of the above observations demonstrate that AgNPs provide a compelling alternative to antibiotics and neither trigger multidrug resistance nor speed up resistance mutation generation. Moreover, AgNPs might be potentially antimutagenic, as inferred from Fig. 4A, even though there is no significant difference between the control and the AgNPs treatment due to the lack of statistical power. Despite this, strict regulations should still be applied to the production, discharge, and disposal of such metal nanomaterials because they could still efficiently select for existing resistant organisms, especially those mutated in the two-component systems mentioned above. Also, it remains a question as to whether metal nanoparticles are mutagenic to eukaryotic genomes, such as target fungal pathogens, or human consumers. A running study of ours may give one answer to this, using the fission yeast Schizosaccharomyces pombe, of which the genome is ~30% homologous to the human genome. A thorough evaluation of the mutagenicity and resistance mechanisms of various metal nanomaterials will facilitate policy-making regarding the environmental risks and usage safety of these high-tech and promising antimicrobial agents.

## MATERIALS AND METHODS

### Preparation and characterization of AgNPs.

To prepare the reducing solution, we followed Hebeish et al. (45). Five grams of native maize starch was dissolved in 80 mL distilled water containing 1.5 g sodium hydroxide at pH 12 and stirred by a magnetic bar at maximum speed. Until starch was dissolved completely, the silver nitrate solution (4.72 g AgNO_3_ dissolved in 20 mL distilled water), sterilized by filtration (Millex; catalog no. SLGP033RB), was added to the reducing solution drop by drop, and then the mixture was incubated at 70°C and stirred at maximum speed in a magnetic stirrer for 60 min. We then cooled the mixture to 25°C, added an equal volume of absolute ethanol, and rinsed the products by centrifuging at maximum speed at 25°C. We repeated the ethanol addition and centrifugation twice and air-dried the mixture.

To evaluate the stability and surface charge of AgNPs in liquid, we measured zeta potential using a size analyzer (Malvern Panalytical Zetasizer Nano ZS90). Each sample was diluted to 60 μg/mL with distilled water and measured for ~5 s three times from −100 to 100 mV. The absorption spectroscopy of reduced AgNPs (1:100 diluted by distilled water with a resistivity of 16 MΩ/cm at 25°C) was measured by a Unico UV-2100 spectrophotometer at wavelengths between 250 and 600 nm. The size and shape were measured by a Jeol transmission electron microscope (JEM-1200EX) with an acceleration voltage of 100 kV, after drying a drop of aqueous AgNPs on a carbon-coated copper TEM grid. The surface traits of nanoparticles were observed by a Carl Zeiss Merlin scanning electron microscope. We measured the pH of agar plates containing AgNPs by using a portable pH meter (Thermo Scientific Orion Star A121).

### Strain, culturing, and determination of the AgNPs’ MICs for E. coli.

Escherichia coli MG1655 was from Patricia Foster’s lab, Indiana University at Bloomington. LB agar or broth (Solarbio; catalog nos. L1015 and L8291, respectively) was used for cell culturing during transferring, freezing, and DNA extraction. To determine MICs, ~1,000 cells of E. coli were incubated on LB plates with gradient concentrations of AgNPs (0, 20, 40, 60, 80, and 100 μg/mL). The efficiency of plating (EOP) was then calculated by EOP = *m*/*N*, where *m* is the CFU of the plate treated with AgNPs, and *N* is the CFU of the blank control plate without AgNPs.

### MA transfers, experimental evolution, and resistance testing.

One single colony of E. coli was propagated and used as the ancestor of all of our experiments (Fig. 1). The 400 MA lines initiated were single colony transferred, and the cell divisions were estimated by CFU every 30 days. Each set of 200 lines were transferred daily on LB agar or LB agar with 60 μg/mL AgNPs at 37°C. Each MA line on LB or LB-AgNPs agar underwent 40 transfers; each transferring cycle took approximately 27.48 and 27.81 cell divisions, respectively (~1,099 and 1,112 total cell divisions for each MA line, respectively).

Acute treatment of E. coli on LB agar with 480 μg/mL AgNPs was used for selecting resistant colonies. The ancestral strain was resuscitated and serially diluted in 1× phosphate-buffered saline (PBS) buffer (10 mM). An average of 36 cells were inoculated into each of 30 test tubes and cultivated to an OD of ~1. Then, ~1 × 10^5^ cells were plated onto LB agar containing 480 μg/mL AgNPs to select resistant bacteria. To collect mutants with diverse genotypes, we only selected one colony from one test tube and then cultured, froze, extracted DNA, and sequenced with mean depth of coverage of 78.93× (SD = 49.69) and mean mapping rate of 99.89% (SD = 0.0027). To avoid batch effect, we also performed the resistant screening at two different time points. Thirty-five and 40 resistant bacteria were selected for the first and second batches, respectively.

Twenty-four parallel populations for experimental evolution, originated from a single ancestral cell, were cultured in 100 mL LB broth in 250-mL flasks, and these included 12 control lines (evoLL1 to evoLL12) and 12 treated lines with 60 μg/mL AgNPs (evoLS1 to evoLS12). We transferred 0.1 mL of each culture into 99.9 mL of fresh medium every 24 h, and they were incubated at 37°C at 200 rpm for 40 days. The mean final cell densities of the evolved control and treatment (measured by CFU) were 2.75 × 10^9^/mL and 2.82 × 10^9^/mL, respectively (SDs, 0.26 × 10^9^ and 0.20 × 10^9^, respectively), which represent ~10 cell divisions per culturing cycle. Cross-contamination was strictly prevented by using dedicated containers, tools, operators, and so on.

Resistance tests followed Long et al. (38). Cells were cultured for 16 h to an OD at 600 nm (OD_600_) of ~1.5 at 37°C, and then about 1,000 cells were plated onto LB plates with AgNPs (0, 1/4×, 1/2×, 1×, 2×, 4×, and 8× MIC of AgNPs). After 24 h, the mean resistance in MIC of the three replicates for each tested line was calculated by colony counting.

### Nucleic acid extraction, library construction, and sequencing.

All of the genomic DNA of E. coli from MA, mutant selection, and experimental evolution was extracted with Wizard genomic DNA purification kit (Promega, Madison, WI). The DNA libraries of E. coli were constructed using a modified protocol for TruePrep DNA library prep kit v2 for Illumina (catalog no. TD501-1; Vazyme Biotech Co., Ltd.) by following Li et al. (98), and Illumina PE150 sequencing was performed on a NovaSeq 6000 machine of Berry Genomics, Beijing.

In order to compare the differential gene expression in the control and the sublethal AgNPs treatment, colonies of E. coli were grown at the same conditions as MA: ancestor cells were streaked onto LB plates and incubated at 37°C for 16 h, and single colonies were then randomly picked and streaked onto LB plates with or without AgNPs and cultured at 37°C. Cells on each plate were then collected by rinsing with cold double-distilled water (ddH_2_O) and transferred into Eppendorf tubes. Four replicates were prepared for the control or the AgNPs treatment. Total RNA was then extracted by Epicentre MasterPure complete DNA and RNA purification kit (catalog no. MC85200). The concentration was quantified via a Qubit 3.0 fluorometer, and purity was measured with a microvolume spectrophotometer (Allsheng Nano-300). The cDNA library was constructed with Ribo-off rRNA depletion kit for bacteria (Vazyme; catalog no. N407-02) and VAHTS mRNA-seq V3 library prep kit for Illumina (catalog no. NR611-01), and Illumina PE150 sequencing was performed on a NovaSeq 6000 sequencer of Berry Genomics, Beijing.

### Identification of mutations.

For MA lines, after trimming adaptors and filtering low-quality reads by using fastp (v0.20.0) (99), the clean reads of E. coli MA lines were mapped to the reference genome of Escherichia coli MG1655 (GenBank accession no. NC_000913.3) by using Burrows-Wheeler Aligner (v0.7.17)-MEM (100). We used the HaplotypeCaller module of Genome Analysis Toolkit (GATK v4.1.2) to call SNPs and small-scale indels using standard hard filters (101–103) and validated candidate calls by Integrative Genomics Viewer (104). The mean mutation rate (μ) was calculated by the formula $\text{μ}=\frac{m}{\sum_{1}^{n}N\times T}$, where *m* is the total number of mutations observed in all MA lines, *n* is the number of MA lines, and *N* and *T* are the total analyzed sites and number of cell divisions passed in each MA line, respectively. The standard error of the mean (SEM) of the mutation rate was calculated by the formula $\;\text{SEM}=\frac{\text{SD}}{\sqrt{n}}$, where SD is the standard deviation of the line-specific mutation rates. The confidence intervals of mutation rate estimates were calculated using the Poisson cumulative distribution function approximated by the χ*^2^* distribution. The mutations of the acutely screened resistant bacteria were called by the same method as MA lines.

For the experimental evolution, the processes of trimming and filtering raw data were the same as described above, and then the clean data were mapped to the same reference by using Bowtie2 (v2.2.5) (105). The calling of population-level mutations was performed by breseq (v0.35.7) via the –p parameter (106). Besides the three default filters, read alignments (RAs), missing coverage (MC), and new junction (NJ), we also filtered out 10 sites which were shared in all of the control and treated lines and resulted from the differences between the ancestral and reference genomes.

### GO and KEGG analyses of differential gene expression.

RNA-seq data were first filtered by fastp (v0.20.0), and the clean data were mapped to the reference and converted to SAM files by HISAT2 (v2.1.0) (107). SAMtools (v1.9) was used to convert the SAM files to BAM and to sort the BAM files, and the matrix of gene expression was generated by using featureCounts (v1.6.4) (100, 108, 109). The DGE analysis was performed with the R package DESeq2, and significance was determined by adjusted *P* value of >0.01 and fold change of <2 (110). GO and KEGG analyses were performed with clusterProfiler (v4.0), and the filtering parameters were pAdjustMethod of BH, pvalueCutoff of 0.05, and qvalueCutoff of 0.05.

### Knockout of *cusS* and *arcA*.

We followed the Red/ET recombineering method developed by Zhang et al. (111). Briefly, we transformed the plasmids pSC101 with the Red/ET recombinase into the E. coli strains by electroporation. Then, two ~50-bp flanking sequences (primers are listed in Table S17 in the supplemental material) in the gene were selected as homology arms and then ligated to the ends of the selection marker gene (chloramphenicol resistance gene) by PCR. PCR products were introduced into the cells expressing Red/ET recombinase (expression induced by rhamnose) and recombined to knockout *cusS* and *arcA*. We also confirmed that the marker gene had no effect on AgNPs resistance.

### Data availability.

All Illumina sequences were uploaded to NCBI BioProject under accession no. PRJNA551791, study no. SRP222431 in SRA.
