# Supplementary material for: Mutagenesis and Resistance Development of Bacteria Challenged by Silver Nanoparticles
Source: Antimicrob Agents Chemother. 2022 Sep 12;66(10):e00628-22. doi: 10.1128/aac.00628-22 (PMC9578424; doi:10.1128/aac.00628-22)
Supplement: Supplemental file 1 — Supplemental methods and Fig. S1 to S6. Download aac.00628-22-s0001.pdf, PDF file, 1.6 MB [file aac.00628-22-s0001.pdf]

Supplementary Methods

Ka/Ks calibration

Ka/Ks, the ratio of the nonsynonymous substitution rate to the synonymous rate, estimates the selective strength conferred on a gene. However, in our experimental evolution experiments (control or 60 μg/ml AgNPs treatment), many genes had 0 synonymous mutation hits, i.e. the denominator Ks being 0. We thus calibrated the ratio by modeling the synonymous base substitutions in each gene of either the control or the treatment, by taking mutation rates from MA transferred on plain LB into account and assuming that synonymous sites are neutral. Thus, the expected number of synonymous substitutions is modelled by:

$$\tau(d) = \sum_{1}^{n} N\mathrm{m}\,\mu\mathrm{d}$$

Where *n* is the number of transfers, *N* is the number of cell divisions per transfer, *m* is the number of parallel lines in either group, μ is the mutation rate at four-fold degenerate sites of MA without AgNPs treatment, *d* is the number of synonymous sites in the gene.

We evaluated the goodness of fit with the observed synonymous substitutions in each gene of each group, using a regular linear model: $y = \alpha + \beta x$, where *x* is the number of four-fold degenerate sites in the gene, y is the observed number of synonymous substitutions. Based on regressions of the observed data, we set the limits of $\alpha\ (-3, 3)$ and $\beta\ (-0.01, 0.01)$ and then performed 1000 random bootstrappings of their combinations (fig. S3B, Supplementary Material), and compared the expected Ks based on the top ten best-fit α and β combinations with our Ks estimates ($\sum_1^n \mathrm{Nm}\,\mu$), using the least-squares method. We also used another simple method to check modelled Ks, assuming $\mathrm{Ks} = \frac{Ms}{Ns} \approx \frac{Ms+1}{Ns}$, where the *Ms* is synonymous substitutions and *Ns* is synonymous sites and the result is similar to the above. Our Ks in the final Ka/Ks calculation for each gene was based on the modeling.

Supplementary Figures

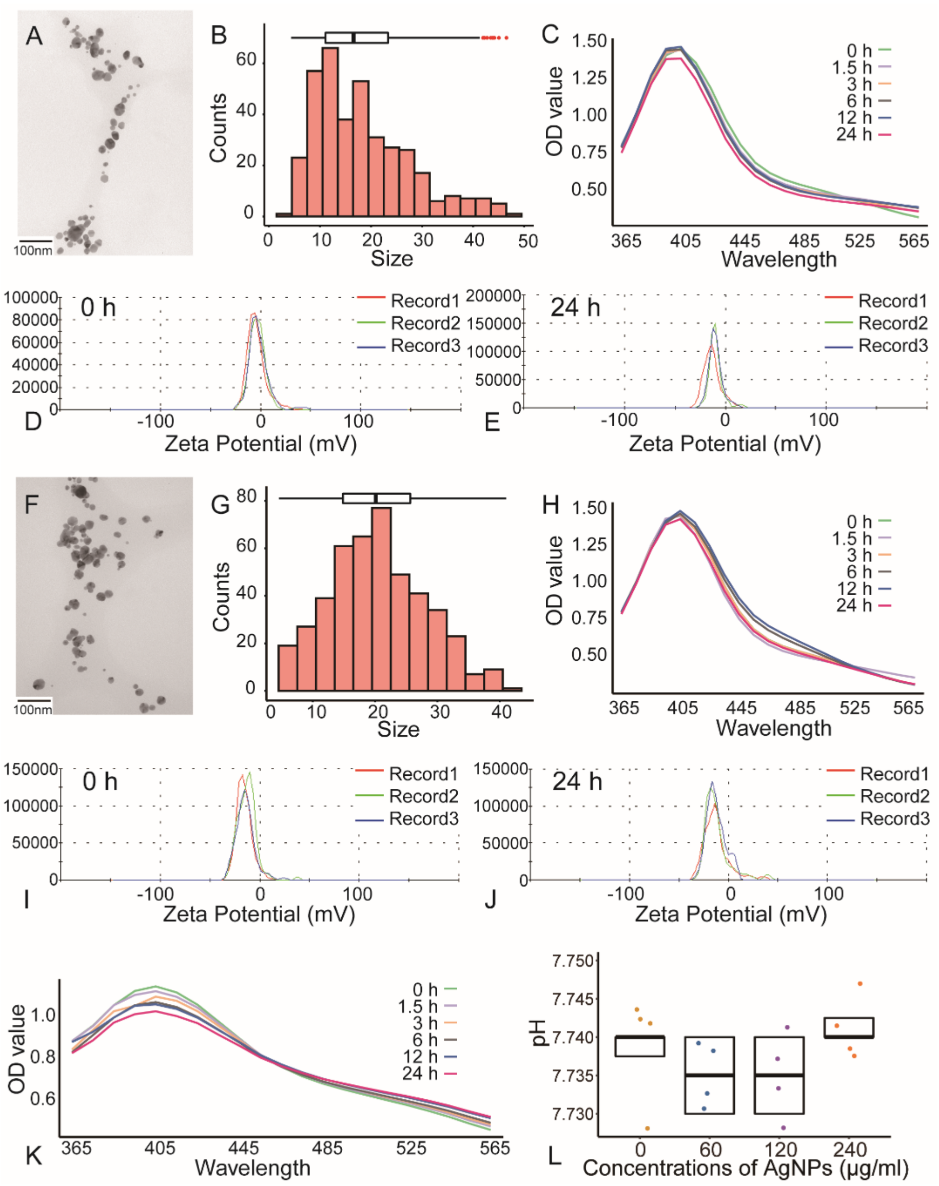

Fig. S1. The physical characteristics of AgNPs at 25 ℃ (A-E, L), 60 ℃ (F-J; water-bathed for 10 minutes, mimicking the process of adding AgNPs to 60 ℃ hot agar when making LB plates with AgNPs) and in LB broth (K). (A) The TEM of AgNPs. **(B)** The size distribution of AgNPs, based on 373 AgNPs. The boxplot shows the median size ~18.21 nm (SD = 9.28). **(C)** The absorbance of UV-spectrum measured by UV-vis at 0h, 1.5h, 3h, 6h, 12h, 24h. **(D, E)** The zeta potential of AgNPs at 0h and 24h respectively and the mean zeta potential is -15.27 mV (SD = 0.19) and -13.70 mV (SD = 0.50). **(F)** AgNPs under TEM. **(G)** Size distribution of AgNPs with the median of 20.46 nm (SD = 7.62; 452 AgNPs were measured). **(H)** UV-spectrum in distilled water within 24h. **(I)** The zeta potential of AgNPs (-14.97 mV, SD = 0.66) at 0h and **(J)** the zeta potential (-13.36 mV, SD = 0.26) at 24 h at 60 **℃. (K)** UV absorbance time-series of AgNPs in LB broth. **(L)** pH of LB plates with different concentrations of AgNPs (0, 60, 120, 240 μg/ml).

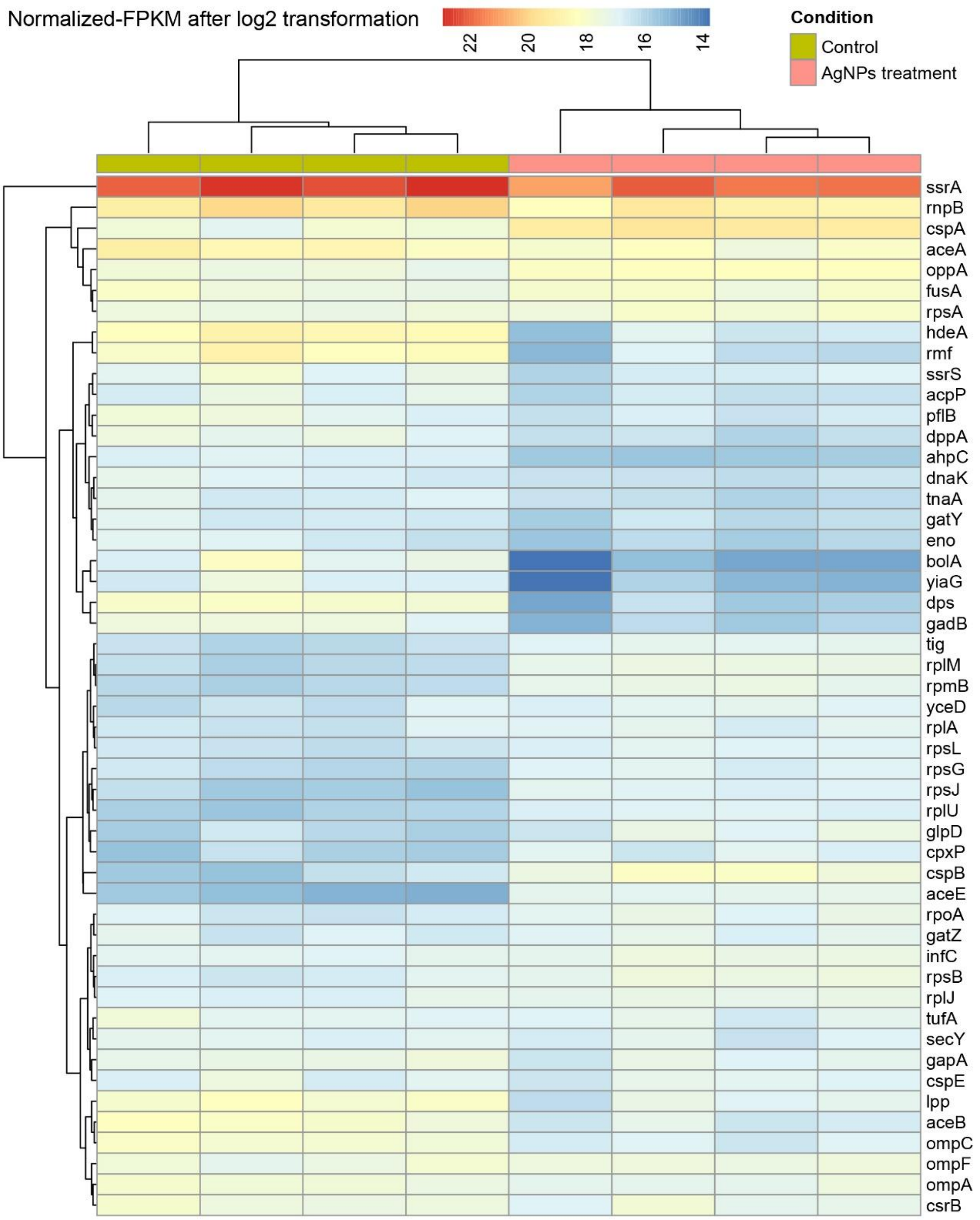

Fig. S2. The heat map of top 50 differentially-expressed genes in the control and the AgNPs-treatment after log2 transformation.

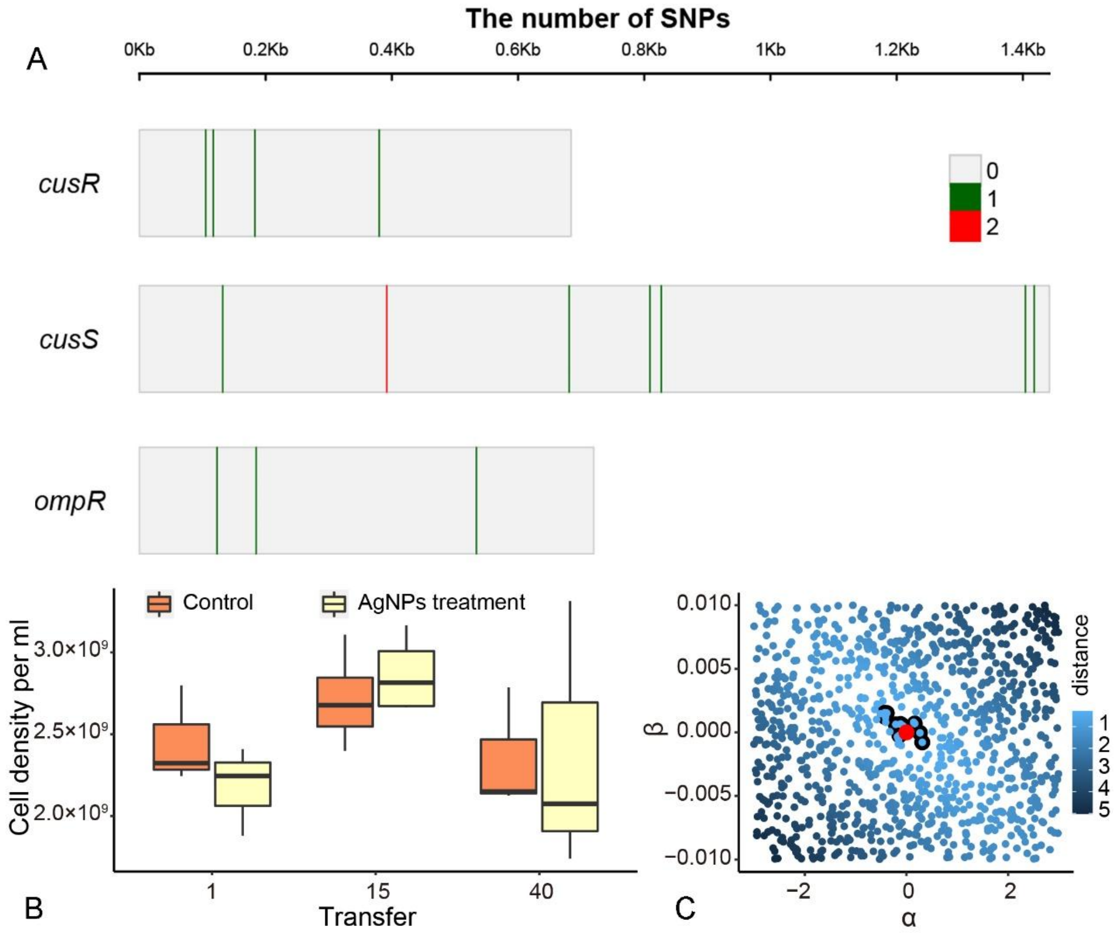

Fig. S3. (A) The SNP density of resistance genes—*cusR*, *cusS* and *ompR*. **(B)** The CFU of the control and AgNPs-treatment in the 1st, 15th and 40th transfer respectively. **(C)** The expected vs. the mean Ks. Blue dots represent the expected Ks from linear regression model, and dots in black are the top ten best fits. The red dot represents the mean Ks from MA.

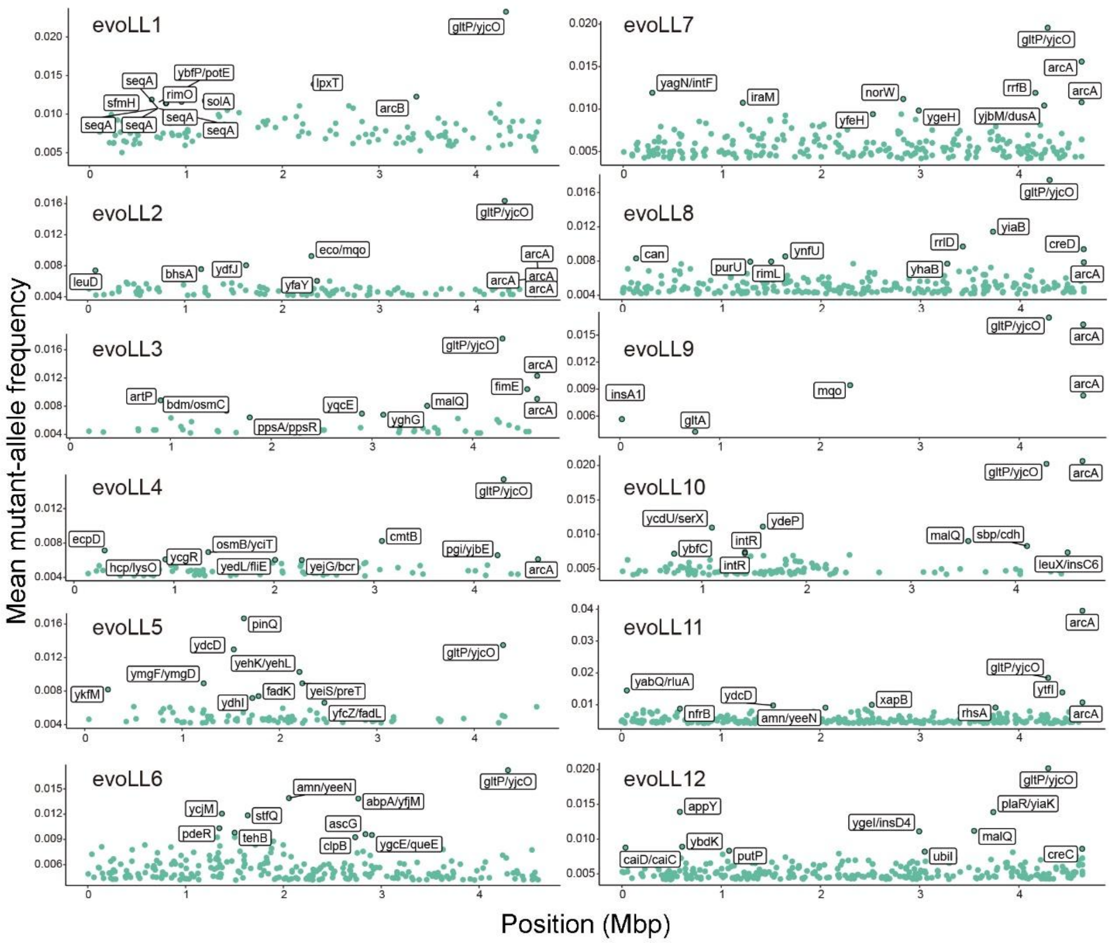

Fig. S4. The mean mutant-allele frequencies of genomic sites in the control lines.

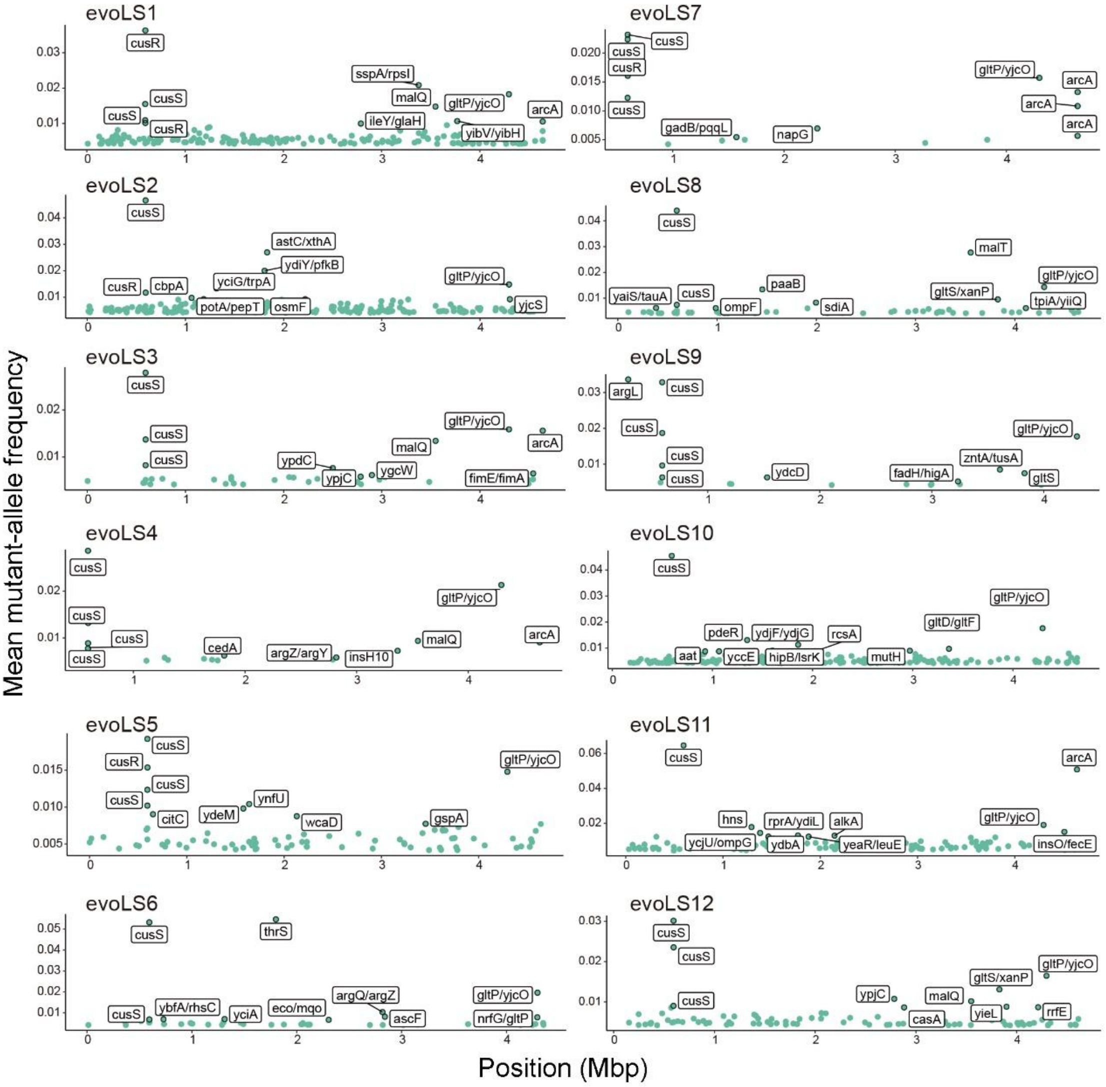

Fig. S5. The mean mutant-allele frequencies of genomic sites in the AgNPs-treated lines.

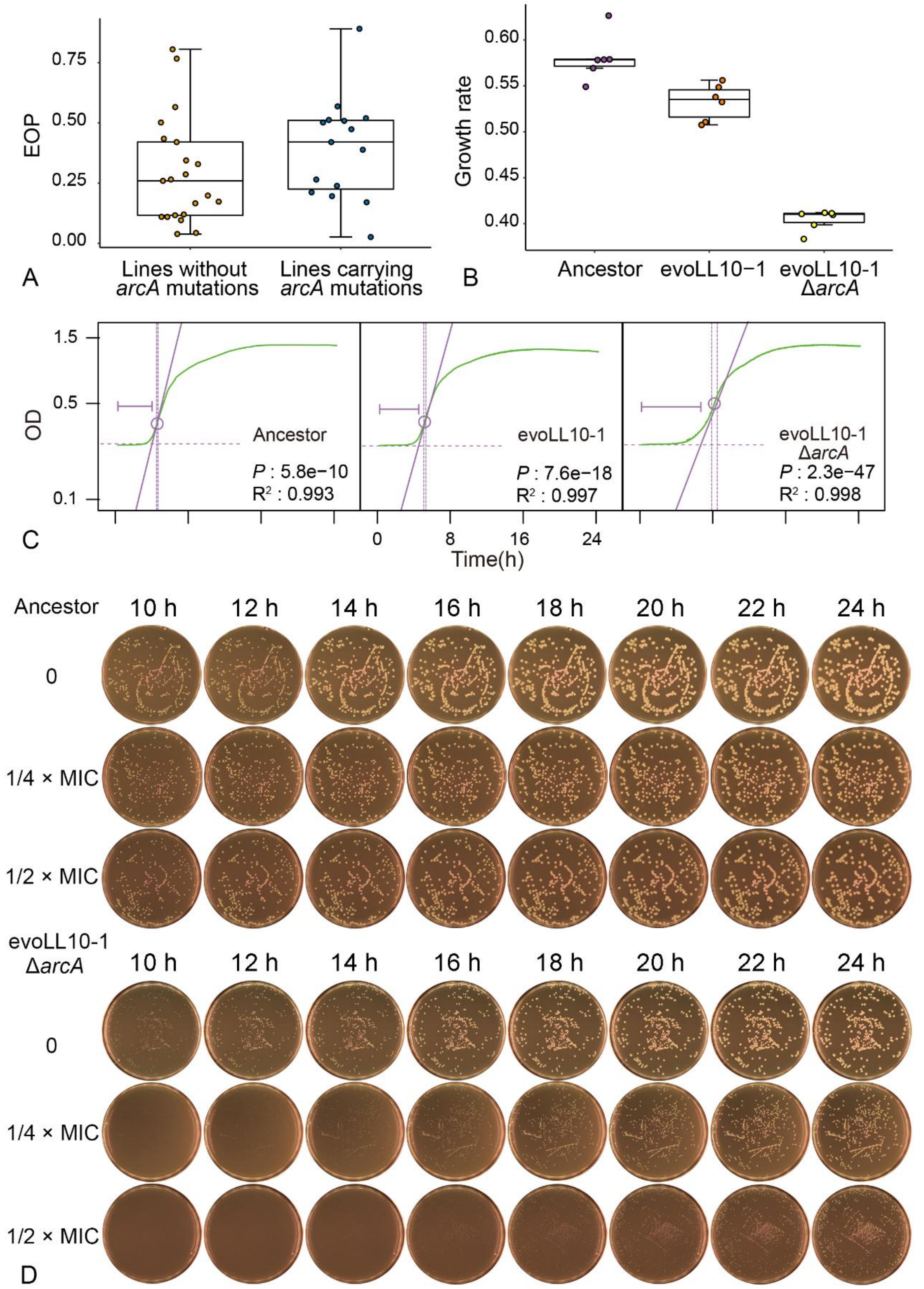

Fig. S6. The EOP (A) and growth rates (B-D) of the control experimental evolution lines. **(A)** The lines were from single colonies of the control experimental evolution lines; the EOP of these lines with and without *arcA* mutations at 2 × ancestral MIC. **(B)** The growth rates of ancestor, evoLL10-1 and evoLL10-1 Δ*arcA* in LB broth without AgNPs. **(C)** From left to right is the bacterial growth curve of the ancestor, evoLL10-1 and evoLL10-1 Δ*arcA*. Horizontal purple line segments represent duration of the lag phase. The purple circles mark the time points

of the maximum growth rates during exponential phase. **(D)** Colony sizes of the ancestor and evoLL10-1 Δ*arcA* from 10 to 24 hours, treated with 0, 1/4× or 1/2× MIC of AgNPs. Note colonies are tiny on the evoLL10-1 Δ*arcA* AgNPs-treated plates.
